# Investigation of SNP markers for the melatonin production trait in the Hu sheep with bulked segregant analysis

**DOI:** 10.1186/s12864-023-09494-z

**Published:** 2023-08-30

**Authors:** Hao Wu, Wenkui Ma, Laiqing Yan, Fenze Liu, Shang Xu, Pengyun Ji, Shuai Gao, Lu Zhang, Guoshi Liu

**Affiliations:** 1 National Engineering Laboratory for Animal Breeding, Key Laboratory of Animal Genetics and Breeding of the Ministry of Agricultural, Beijing Key Laboratory for Animal Genetic Improvement, College of Animal Science and Technology, China Agricultural University, No 2, Yuanmingyuan West Road, Beijing, 100193 China; 2 Sanya Institute of China Agricultural University, Sanya, 572025 China; 3 Hainan Yazhou Bay Seed Laboratory, Sanya, 572025 China; 4 Inner Mongolia Golden Grassland Ecological Technology Group Co., LTD., Bayannaoer, 015000 China

**Keywords:** Hu sheep, Melatonin, Bulked segregant analysis, *AANAT*, *ASMT*, SNP

## Abstract

**Background:**

As an important reproductive hormone, melatonin plays an important role in regulating the reproductive activities of sheep and other mammals. Hu sheep is a breed favoring for meat, with prolific traits. In order to explore the relationship between melatonin and reproductive function of Hu sheep, 7,694,759 SNPs were screened out through the whole genome sequencing analysis from high and low melatonin production Hu sheep.

**Results:**

A total of 68,673 SNPs, involving in 1126 genes, were identified by ED association analysis. Correlation analysis of SNPs of *AANAT*/*ASMT* gene and *MTNR1A*/*MTNR1B* gene were carried out. The melatonin level of CG genotype 7,981,372 of *AANAT*, GA genotype 7,981,866 of *ASMT* and GG genotype 17,355,171 of *MTNR1A* were higher than the average melatonin level of 1.64 ng/mL. High melatonin Hu sheep appear to have better multiple reproductive performance.

**Conclusions:**

By using different methods, three SNPs which are associated with high melatonin production trait have been identified in Hu sheep. These 3 SNPs are located in melatonin synthetase *AANAT*/*ASMT* and receptor *MTNR1A*, respectively. Considering the positive association between melatonin production and reproductive performance in ruminants, these three SNPs can be served as the potential molecular markers for breading Hu sheep with the desirable reproductive traits.

**Supplementary Information:**

The online version contains supplementary material available at 10.1186/s12864-023-09494-z.

## Introduction

Small ruminants, particularly sheep significantly impact the daily life of a considerable part of human population socio-economically [1]. These small ruminants provide meat, wool, skin and other products for our consuming. To select the breed with high quality meat [2], wool fineness [3], large number of offspring [4] and carcass weight [5] becomes the urgent agenda for researchers. Hu sheep (*Ovis aries*) is a unique sheep breed in China. This breed has the advantages of early sexual maturity, strong fertility with large litter size and easy adapts to coarse feeding materials [6]. By analyzing its reproductive traits with genome-wide association study it was found that the polymorphisms of *FecB*, *GDF9* and *TGFBR2* were significantly associated with the fertility trait of Hu sheep [7] and these genes might be the potential marker sites for breeding selection. As the advancement of molecular breading technology, the combined trials with emphasis on administration and genetic progress to improve the animal breading outputs have shown their decisive significance [8]. Specific to sheep, to improve productivity and reproductive performance of ewes with economical and biological efficiency has become the goal of sheep production enterprises [9].

Melatonin is an important reproductive hormone which is synthesized in many tissues and organs including the pineal gland [10] and reproductive system [11]. Melatonin modulates the release of follicle-stimulating hormone (FSH) and luteinizing hormone (LH) through the hypothalamic-pituitary–gonadal axis thus regulating the seasonal reproductive activities of mammals [12, 13]. It also improves oocyte quality through its antioxidant function [14]. It was found that before the *MTNR1A* expression peak prior to ovulation, melatonin application could improve luteal function, increase progesterone secretion level, pregnancy rate and litter size of animals [15]. It appears that melatonin plays an important role in animal reproduction.

Single nucleotide polymorphism (SNP) mainly refers to DNA polymorphism at the genome level, which is characterized by large number, wide distribution and genetic stability [16]. Currently, SNPs are widely used in genome-wide association, germplasm resource and genome selection signal analyses. Gene chips developed based on SNP typing technology have been mainly used for genetic resource mining and breeding selection [17]. Bulked segregant analysis (BSA) method is also a method based on SNP analysis, which can rapidly locate candidate genes for extreme traits [18]. Bulked segregant analysis (BSA) has been widely used in molecular marker screening and Quantitative trait locus (QTL) localization analysis of target traits in plants and animals [19, 20].

In this study, BSA method will be used to analyze the genotypes of individual Hu sheep to match up their endogenous melatonin levels. The focus will be given to the SNP polymorphisms of *AANAT*/*ASMT* as well as *MTNR1A*/*MTNR1B*. Our purpose is to identify whether SNPs among these genes are associated with melatonin production in Hu sheep.

## Materials and methods

### Sample collection

In the current study, Hu sheep were selected to investigate whether their excellent reproductive performance was associated with the melatonin production traits by the method of whole-genome SNP level. In order to analyze the melatonin production trait of Hu sheep, the whole genome SNP of Hu sheep who exhibited either high or low melatonin production trait were analyzed. The correlation between the SNPs of enzyme genes for melatonin synthesis, *AANAT*/*ASMT,* and genes of receptors, *MTNR1A*/*MTNR1B,* was also analyzed. If some of these SNPs of melatonin trait are strongly associated with reproductive performance of Hu sheep these SNPs may serve as the biomarkers for guiding the breeding selection of a new strain of Hu sheep. A total of 195 Hu sheep (Information as shown in Table S1) were selected from Inner Mongolia Golden Grassland Ecological Technology Group Co., LTD. For blood collections, the 5 mL of blood was collected from neck veins at eight o 'clock, centrifuged 3,000 rpm for 8 min, serum was taken and stored at -20℃ for future use.

### Melatonin detection

Melatonin standard curve was prepared by serial dilutions of melatonin from 100, 50, 20, 10 and 5 ng/L respectively. Peak area was detected and standard concentration curve was drawn. The 200μL of serum was added with 800μL methanol, swirled for 30 min and centrifuged at 14,000 rpm for 20 min at 4 ℃. The supernatant was filtered by the 0.22 μm filter and cooled down to -20 ℃ then, was detected by Liquid chromatography tandem mass spectrometry (LC–MS/MS) (Agilent1290-G6470, Santa Clara, CA, USA) in the Central Laboratory of Institute of Animal Science, Beijing, Chinese Academy of Agricultural Sciences. The Symmetry C18 column was used to separate the melatonin from the samples.

### Quality control analysis of Illumina HiSeq DNA resequencing data

Blood DNA was extracted following the instructions of the Kit (NEBNext®Ultra™DNA Library Prep Kit for Illumina®) for library construction. A sample of 1 μg genomic DNA was randomly broken into fragments < 500 bp by ultrasonic treatment (Covaris S220). End repair was performed on the fragment with End Prep Enzyme Mix, appropriate ends were added to both ends with T-A ligase, then 5 'phosphorylation and da-tailed were performed. Adaptor-ligated DNA was size-screened using the AxyPrep Mag PCR cleanup (Axygen) to obtain fragments about 410 bp. Each sample was amplified by PCR using P5 and P7 primers for 8 cycles, respectively. Both P5 and P7 primers carry bridge PCR sequences that can be annealed by flow cytometers. The P7 primer carries a six-base index for multiplexing. PCR products were cleaned using AxyPrep Mag PCR (Axygen) and validated using Agilent 2100 Bioanalyzer (Agilent Technologies, PaloAlto, CA, USA). Quantification was performed using Qubit 2.0 fluorometer (Invitrogen, Carlsbad, CA, USA). According to the kit instructions (Illumina, San Diego, CA, USA), libraries with different indexes were multiplexed and loaded onto the Illumina HiSeq instrument. Sequencing was performed using the 2X150 paired terminal (PE) configuration. Using HiSeq control software (HCS) + OLB + gappeline-1.6 (Illumina) for image analysis and basic invocation on HiSeq instrument.

The bcf2fastq (version 2.17.1.14) software was used for image Base calling of original image Data. Pass Filter Data (PF) in FASTQ (fq) format was obtained. Clean Data was obtained by using cutadapt (version 1.9.1) software to remove junctions and low-quality sequences from Pass Filter Data. Clean data was compared to reference Genome sequences using Dragen Genome Pipline. For the resulting BAM (binary SAM file) file, Picard and GATK were used to remove duplicate data of PCR and collect the local realignment, base quality recalibration and the corrected genome alignment results. The coverage and coverage depth of the genome were calculated according to the comparison results.

### SNP detection and annotation

According to the comparison results of Clean Reads in reference genome, the Haplotype Caller module of software GATK (version 4.0.4.0) was used for mutation detection. Filtration was done using the Variant Filtration module, Filter parameters for:—filter Expression “QD < 2.0 | | MQ < 40.0 | | FS > 60.0 | | SOR > 3.0 | | MQ Rank Sum < 12.5 | | Read Pos Rank Sum < -8.0”. ANNOVAR software was used for the functional annotation of the detected gene variants. When the coverage depth of the sample at a SNP site is < 5X, the site is considered as missing and listed as NA in the table. When the sample genotype mutation frequency at a SNP site is ≥ 0.8 or ≤ 0.2, the site is a pure sum. If the mutation frequency is between 0.2 and 0.8 and each allele of the heterozygous genotype has at least 4 reads, the SNPs is considered as the heterozygous mutation; otherwise, the site is treated as deletion and listed as NA in the table. Finally, all SNPs of genotypes in the tested samples were summarized in the table for comparison.

### SNP detection and annotation

Euclidean Distance (ED) algorithm was used to search for significantly different markers between pools. In the process of ED analysis, MMAPPR software package will used to filter SNP loci of non-secondary gene and SNP loci with sequencing depth less than 10X of wild type or mutant mixed pool. If the frequency of A, T, C and G in the wild type pool is greater than or equal to 95%, it will be filtered. The calculation formula of ED method is as follows:

$$ED=\sqrt{{\left({A}_{mut}-{A}_{wt}\right)}^{2}+{\left({C}_{mut}-{C}_{wt}\right)}^{2}+{\left({G}_{mut}-{G}_{wt}\right)}^{2}+{\left({T}_{mut}-{T}_{wt}\right)}^{2}}$$

Note: Amut is the frequency of base A in mutant mixing pool, Awt is the frequency of base A in wild type mixing pool; Cmut is the frequency of C base in mutant mixing pool and Cwt is the frequency of C base in wild type mixing pool. Gmut is the frequency of G base in mutant pool and Gwt is the frequency of G base in wild type pool. Tmut is the frequency of T base in mutant mixing pool and Twt is the frequency of T base in wild type mixing pool.

In this project, the 4th power of the original ED served as the correlation value to eliminate background noise, and this value was used for the local polynomial regression fitting to obtain the fitting curve. The value of Median + 3SD of all locus fitting values was used as the association threshold for analysis. This association threshold was used to determine the correlation area. Thereafter, sites with mutation frequency > 0.75 and Euclidean distance > 0.5 were selected as candidate sites from the correlation area and the annotation results of ANNOVAR were also extracted from these candidate sites.

### GO enrichment analysis

Based on the GO each term mapping database (http://www.geneontology.org/). for candidate genes the numbers of genes in each term were calculated to obtain the genes with a certain GO function and their statistical analysis. Then the GO entries with significantly enriched in the whole genome background were identified by hypergeometric test.

### KEGG enrichment analysis

KEGG enrichment analysis was based on hypergeometric distribution principle [21–23]. Pathway with Qvalue ≤ 0.05 was defined as significantly enriched in candidate genes after multiple test correction. The formula is as follows:

$$P=1-\sum_{I=0}^{m-1}\frac{\left(\begin{array}{c}M\\ \mathrm{i}\end{array}\right)\left(\begin{array}{c}N-M\\ n-i\end{array}\right)}{\left(\begin{array}{c}N\\ i\end{array}\right)}$$

Note: N is the number of genes with Pathway annotation in the whole genome; n is the number of candidate genes screened according to the selection signal index. M is the number of genes annotated for a specific Pathway among all genes; m is the number of candidate genes annotated for a specific Pathway.

### SNP analysis of enzyme genes for melatonin synthesis and genes of melatonin receptors

PCR was carried out for SNP analysis. The related primers were shown in Table 1. The reaction system was composed with 25 μL Premix Taq (TaKaRa Taq Version 2.0 plus dye), genomic DNA (20 ng/µL) 1 μL, primer 1 (20 μM) 1 μL, primer 2 (20 μM) 1 μL, sterilized water Up to 50 μL. PCR procedure: predenaturation 95 ℃ for 5 min, 30 cycles 94 ℃ for 30 s, 60℃ for 30 s, 72 ℃ for 10 s, 72 ℃ for 5 min, stored at 4 ℃. PCR products were sequenced by Jinweizhi Biotechnology Co., Ltd. Sequence results were compared by DNASTAR (version 7.1) and single nucleotide polymorphisms were screened by SeqMan Pro software.

**Table 1:** Primer sequence table

Accession number	Gene	Site	Primer pair	Forward primer(5′-3′) Downstream primer(5′-3′)	PCR product size	Tm
1	AANAT	7,981,745	P01	CATCTCTGTCTCCGGCAACT GAGTCAGCGGTCACTGTTCC	796 bp	59.47 60.67
	AANAT	7,981,372	P02	CATCTCTGTCTCCGGCAACT GAGTCAGCGGTCACTGTTCC	796 bp	59.47 60.67
	AANAT	7,981,866	P03	CATCTCTGTCTCCGGCAACT GAGTCAGCGGTCACTGTTCC	796 bp	59.47 60.67
2	AANAT	7,980,810	P04	TCCTAGAATTTGAGAGCAGGAGTC CCTCTCGCTCAATCTCAAACAC	562 bp	59.60 59.33
3	ASMT	604,657	P05	GTGATGTCTTGAAAAGGAAGACAG AGTATTCCATCGTTCAGAATCGTC	477 bp	58.00 58.72
4	MTNR1B	1,684,394	P06	CTTCTAACCCTGCAGAGCTTCTC CTTCTGCTACCTGCACATCTGG	872 bp	60.68 61.00
	MTNR1B	1,684,464	P07	CTTCTAACCCTGCAGAGCTTCTC CTTCTGCTACCTGCACATCTGG	872 bp	60.68 61.00
	MTNR1B	1,684,557	P08	CTTCTAACCCTGCAGAGCTTCTC CTTCTGCTACCTGCACATCTGG	872 bp	60.68 61.00
	MTNR1B	1,684,269	P09	CTTCTAACCCTGCAGAGCTTCTC CTTCTGCTACCTGCACATCTGG	872 bp	60.68 61.00
5	MTNR1A	17,354,935	P10	CTGACAGCACATTAGCTCAGACAT CCTCTGCTACGTGTTCCTGATCT	774 bp	60.92 61.74
	MTNR1A	17,355,358	P11	CTGACAGCACATTAGCTCAGACAT CCTCTGCTACGTGTTCCTGATCT	774 bp	60.92 61.74
	MTNR1A	17,354,943	P12	CTGACAGCACATTAGCTCAGACAT CCTCTGCTACGTGTTCCTGATCT	774 bp	60.92 61.74
	MTNR1A	17,355,171	P13	CTGACAGCACATTAGCTCAGACAT CCTCTGCTACGTGTTCCTGATCT	774 bp	60.92 61.74
6	MTNR1A	17,377,872	P14	TGAAGGCTTCTTAGTTGGTTCAAG GAGCCGGGGTCATAAAAGG	533 bp	59.18 57.90

### Data analysis

R software was used to analyze the association between SNPs and phenotypes. The analytic model was *Yij* = *μ* + *Gi* + *Pj* + *eij*, *Yij* was the observed value of character;* μ* is the total mean value of character. *Gi* is genotype effect; *Pj* is the age effect; *eij* is random error.

## Results

### Quality control analysis of high-melatonin Hu-sheep sequencing data

Among 195 Hu sheep tested, 100 (51.3%) had melatonin content between 0–0.49 ng/mL, 42 (21.5%) had melatonin content between 0.5- 1.0 ng/mL and 53 (27.2%) had melatonin content above 1 ng/mL. Three individuals with highest and three individuals with lowest melatonin levels were selected for whole genome sequencing analysis (Table 2). The original Data (Pass Filter Data, PF) were shown in Table 3. The average base quality value of sequencing data Reads was concentrated in 36. After removing low-quality sequence results from the original data the Clean Data were obtained as shown in Table 4. The clean data after QC reached more than 99% in PF data, as shown in Table 5. The alignment ratio between clean data and reference genome sequence was 99.11% on average and the unique reads number of reference genome was 81.12%, as shown in Table 6. The average coverage rate reached 96.05% and the average coverage depth reached 28.32%.

**Table 2:** Sample of whole genome sequencing analysis

Number	Melatonin (ng/mL)
B1-1	15.33
B1-2	10.43
B1-3	11.35
B2-1	0.30
B2-2	0.29
B2-3	0.23

**Table 3:** PF data statistics

Sample	Length(bp)	Reads	Bases	Q20(%)	Q30(%)	GC (%)	N(ppm)
B1-1	150.00	691,650,438	103,747,565,700	97.32	92.86	43.87	20.23
B1-2	150.00	664,738,950	99,710,842,500	97.34	97.34	43.91	20.28
B1-3	150.00	711,592,448	106,738,867,200	97.33	97.33	44.09	7.08
B2-1	150.00	705,047,746	105,757,161,900	97.22	92.67	43.87	7.05
B2-2	150.00	743,148,144	111,472,221,600	96.98	92.18	43.88	7.07
B2-3	150.00	655,396,188	98,309,428,200	97.14	92.58	45.67	6.97

(1) Sample: Name of sequencing sample

(2) length: reads average length

(3) Reads: Number of sequencing reads

(4) Bases: Total number of bases

(5) Q20 (%): The percentage of bases with Phred value greater than 20 in the total base was calculated respectively

(6) Q30 (%): The percentage of bases with Phred value greater than 30 in the total base was calculated respectively

(7) GC (%): Calculate the total number of bases G and C as a percentage of the total number of bases

(8) N(ppm): The number of N bases per million that cannot be determined by sequencing

**Table 4:** Clean data Data statistics

Sample	Length(bp)	Reads	Bases	Q20(%)	Q30(%)	GC (%)	N(ppm)
B1-1	149.24	689,571,562	102,911,101,701	97.49	93.07	43.88	7.23
B1-2	149.23	662,659,546	98,886,887,091	97.52	93.19	43.91	7.19
B1-3	149.22	708,955,178	105,790,987,614	97.53	93.16	44.08	5.27
B2-1	149.22	702,403,422	104,813,780,481	97.43	92.93	43.85	5.25
B2-2	149.23	739,557,498	110,365,847,379	97.23	92.47	43.86	5.26
B2-3	149.20	652,649,672	97,377,346,672	97.37	92.85	45.65	5.21

(1) Sample: Name of sequencing sample

(2) length reads: average length

(3) Reads: Number of sequencing reads

(4) Bases: Total number of bases

(5) Q20 (%): The percentage of bases with Phred value greater than 20 in the total base was calculated respectively

(6) Q30 (%): The percentage of bases with Phred value greater than 30 in the total base was calculated respectively

(7) GC (%): Calculate the total number of bases G and C as a percentage of the total number of bases

(8) N(ppm): The number of N bases per million that cannot be determined by sequencing

**Table 5:** Clean data ratio statistics

Sample	PF Reads	Clean Reads	Ratio of Reads (%)	PF Bases	Clean Bases	Ratio of Bases (%)
B1-1	691,650,438	689,571,562	99.70	103,747,565,700	102,911,101,701	99.19
B1-2	664,738,950	662,659,546	99.69	99,710,842,500	98,886,887,091	99.17
B1-3	711,592,448	708,955,178	99.63	106,738,867,200	105,790,987,614	99.11
B2-1	705,047,746	702,403,422	99.62	105,757,161,900	104,813,780,481	99.11
B2-2	743,148,144	739,557,498	99.52	111,472,221,600	110,365,847,379	99.01
B2-3	655,396,188	652,649,672	99.58	98,309,428,200	97,377,346,672	99.05

(1) Sample: Name of sequencing sample

(2) PF Reads: Number of raw data reads

(3) Clean Reads: Number of data reads after QC

(4) Ratio of Reads (%): Clean Reads accounted for the number percentage of PF Reads

(5) PF Bases: Number of bases of raw data

(6) Clean Bases: Number of data bases after QC

(7) Ratio of Bases (%): Clean Bases as a percentage of the number of PF Bases

**Table 6:** Sequencing comparison statistics

Sample	Total Reads	Reads mapped to genome	Mapped Reads Ratio (%)	Uniq Reads	Uniq Reads Ratio (%)
B1-1	689,571,562	688,914,503	99.90	556,777,401	80.74
B1-2	662,659,546	661,975,981	99.90	540,640,059	81.59
B1-3	708,955,178	708,233,372	99.90	569,523,921	80.33
B2-1	702,403,422	701,650,687	99.89	569,634,685	81.10
B2-2	739,557,498	738,574,516	99.87	599,762,726	81.10
B2-3	652,649,672	651,983,170	99.90	534,173,419	81.85

(1) Sample: Name of sequencing sample

(2) Total Reads: The number of all sequencing reads

(3) Mapped Reads: Number of reads with reference genomes for all comparisons

(4) Mapped Reads Ratio (%): The ratio of reads in the reference genome was compared

(5) Uniq Reads: The ratio of reads in the reference genome was compared

(6) Uniq Reads Ratio (%): uniq reads as a percentage of all mapped reads

(5) Mean depth: The average sequencing depth of the covered bases

### Genome-wide SNP detection and annotation

**Fig. 1:**
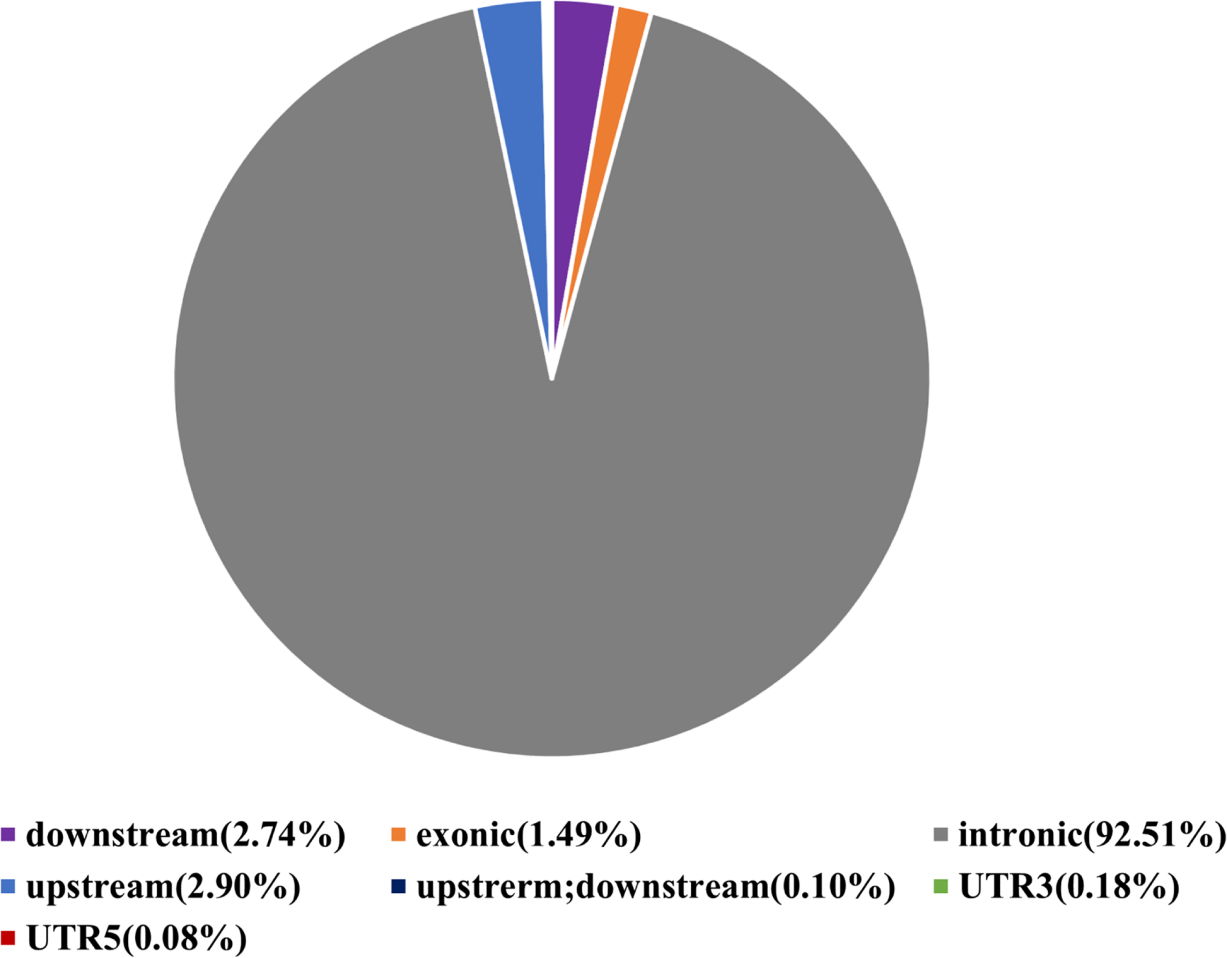
SNP annotation statistics

Based on the comparison results, SNPs were detected using Dragen Genome Pipline. According to the functional changes caused by mutation sites, the proportion of non-synonymous mutations of SNP is higher than that of synonymous mutations (Table 7). SNP mutations mainly focus on C:G > T:A and A:T > C:G. The 7,694,759 SNPs were obtained by screening and filtering the SNPs in the samples. Functional annotation was carried out for the detected variation sites. The variation sites mainly occurred in the intronic and splicing regions of the genome, with a small proportion in the UTR5、UTR3 and UTR5;UTR regions, as shown in Fig. 1.

**Table 7:** Statistics of SNP types in the whole genome

Sample	SNP	Non-Synonymous	Synonymous	Stop gain	Stop lost
B1-1	14,174,904	6860	6183	132	16
B1-2	14,717,257	7069	6419	124	15
B1-3	14,610,225	7161	6498	134	14
B2-1	14,759,244	7258	6618	126	15
B2-2	14,659,280	6722	6304	117	13
B2-3	14,667,510	7278	6592	131	17

(1) Sample: Name of sequencing sample

(2) SNP: Total number of SNPs

(3) Non-Synonymous: Total number of non-synonymous mutations

(4) Synonymous: Total number of synonymous mutations

(5) Stop gain: The number of terminating codons that are mutated (the mutation gives the gene a terminating codon)

(6) Stop lost: The number of stop codon deletions (mutations that cause genes to lose stop codons)

### Analysis of ED method correlation results

**Fig. 2:**
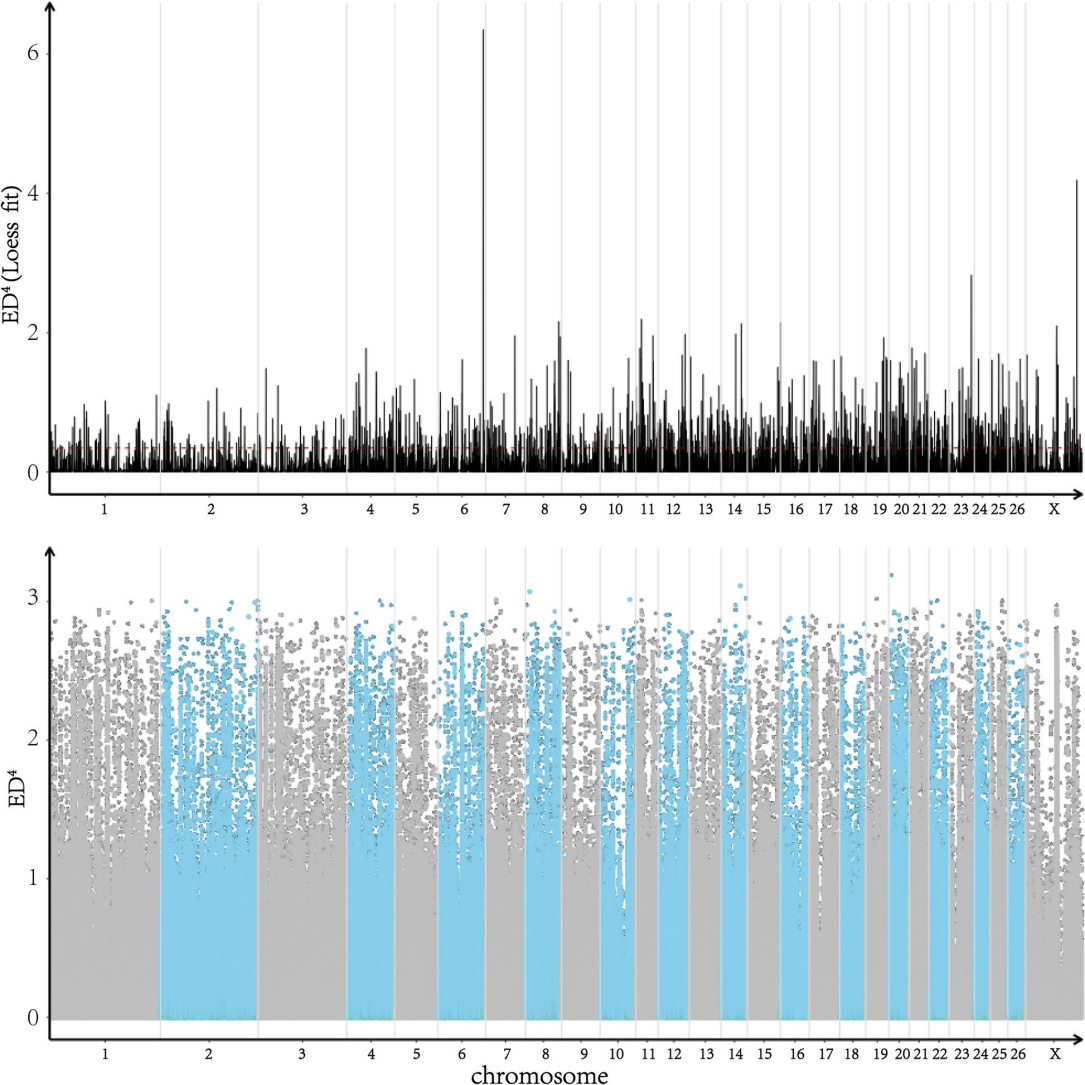
Distribution of ED association values across the genome. The x-coordinate is the name of chromosome, the dot plot represents the ED value of each SNP site, and the line plot represents the ED value after fitting. The higher the ED value, the better the correlation effect of the point, and the blue shadow represents the interval located

Based on ED association method, a total of 3,934,196 SNPs were selected from 7,694,759 SNPs (Table 8). The distribution of these SNPs on each chromosome is shown in Fig. 2. Median + 3SD of all sites fitting values was taken as the association threshold for analysis, and 0.3443 was calculated. According to the correlation threshold, a total of 1111 regions with a total length of 34,805,411 bp were obtained. A total of 68,673 SNPs were selected from the associated regions with mutation frequency > 0.75 and Euclidene distance > 0.5. For the selected SNPs, the annotation results of ANNOVAR were extracted, involving 1126 genes.

**Table 8:** SNP site filtering statistics

Total	Biallelic	Frequency	NA
7,694,759	5,982,601	4,946,276	3,934,196

Total_number: The total number of SNPs

Biallelic: The number of SNPs after filtering non-secondary genes

Frequency: The number of SNPs after the frequency of filter bases is greater than or equal to 95%

NA: The number of SNPs after the sequencing depth of wild type or mutant pool was lower than 10X

### GO enrichment analysis

**Fig. 3:**
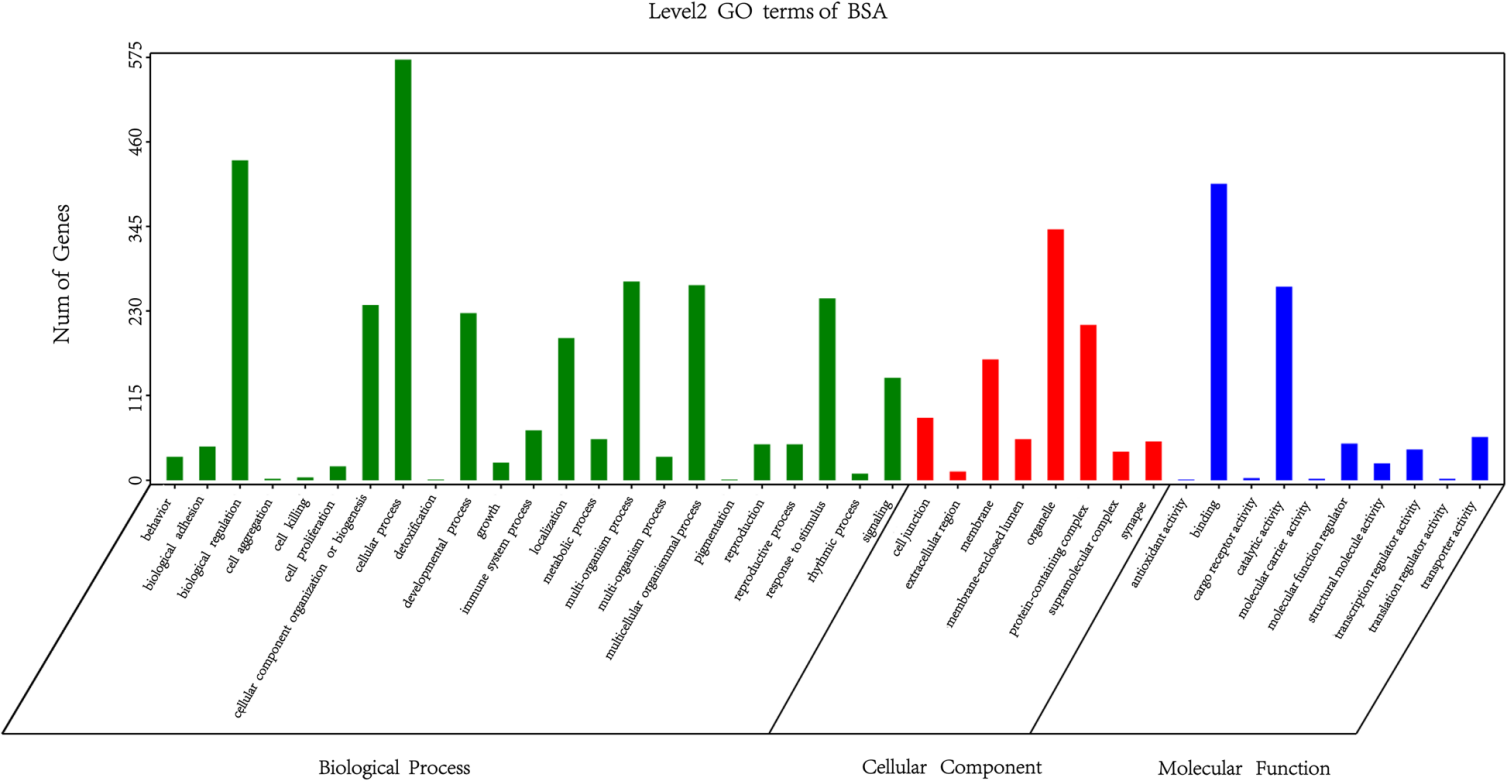
GO functional classification statistics

**Fig. 4:**
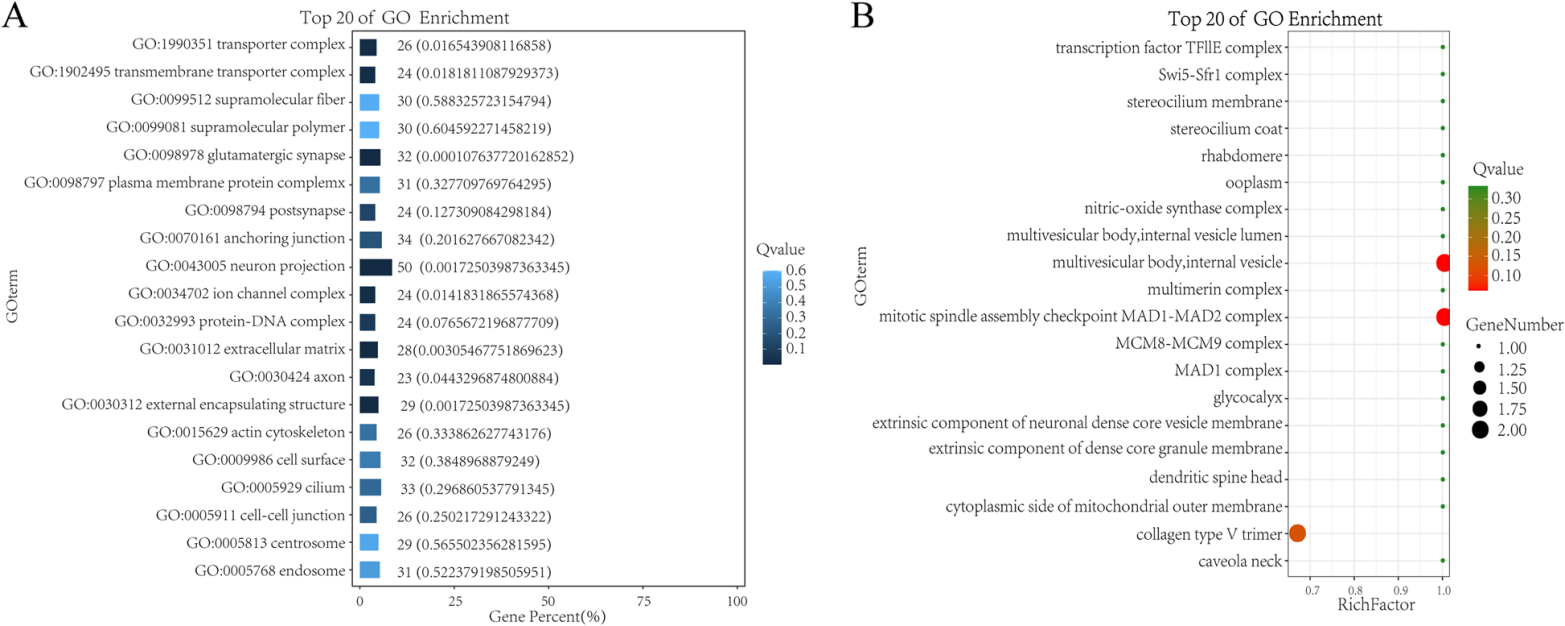
GO enrichment results of cell components. **A** Enrichment bar chart. Note: The ordinate is GO term and the abscissa is the percentage of all differences accounted for by this GO term. From the largest to the smallest, the top 20 are selected. The darker the color, the smaller the Q value. The number of GO term and Q value are labeled above. **B** Bubble chart. Note: The ordinate is GO term, and the abscissa is enrichment factor (the number of differences in the GO term divided by all the numbers). From the largest to the smallest, the top 20 are selected. Size of bubble area: the number of genes belonging to this GO in the target gene set; Bubble color: enrichment significance, that is, the size of Q value; The size is the quantity, and the redder the color, the smaller the Q

**Fig. 5:**
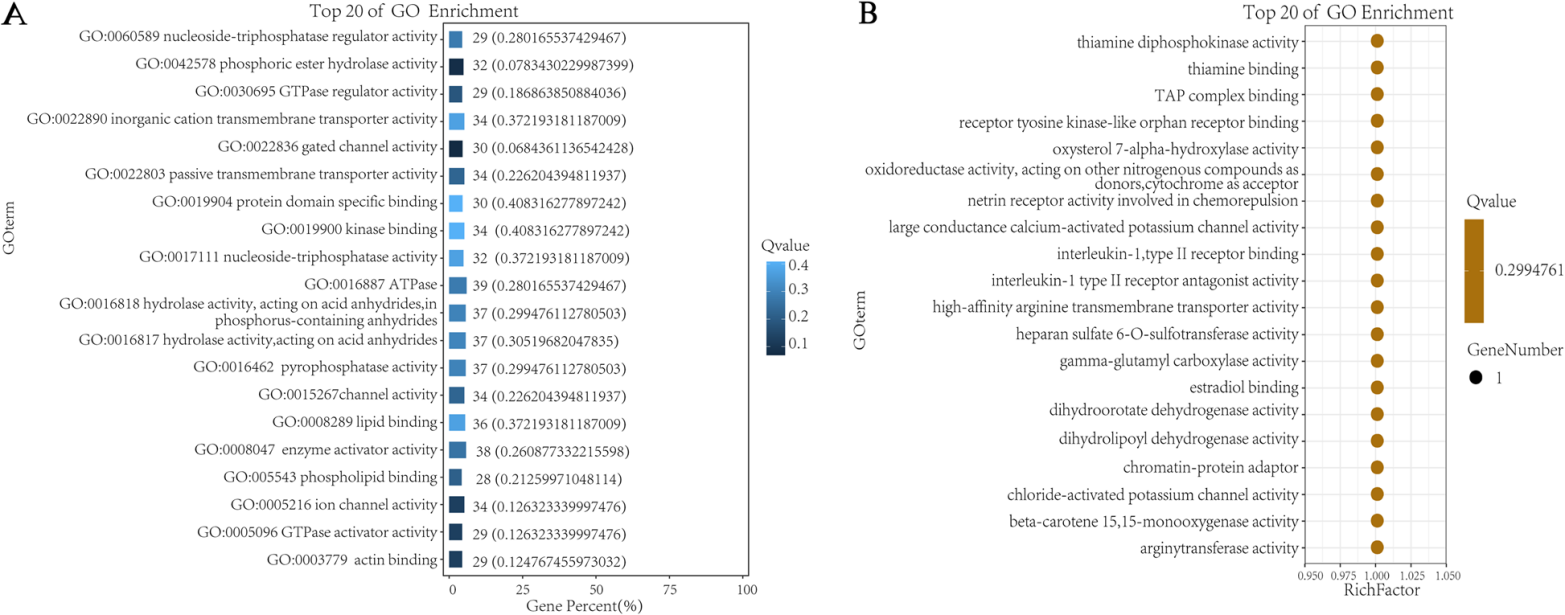
Molecular function GO enrichment results

**Fig. 6:**
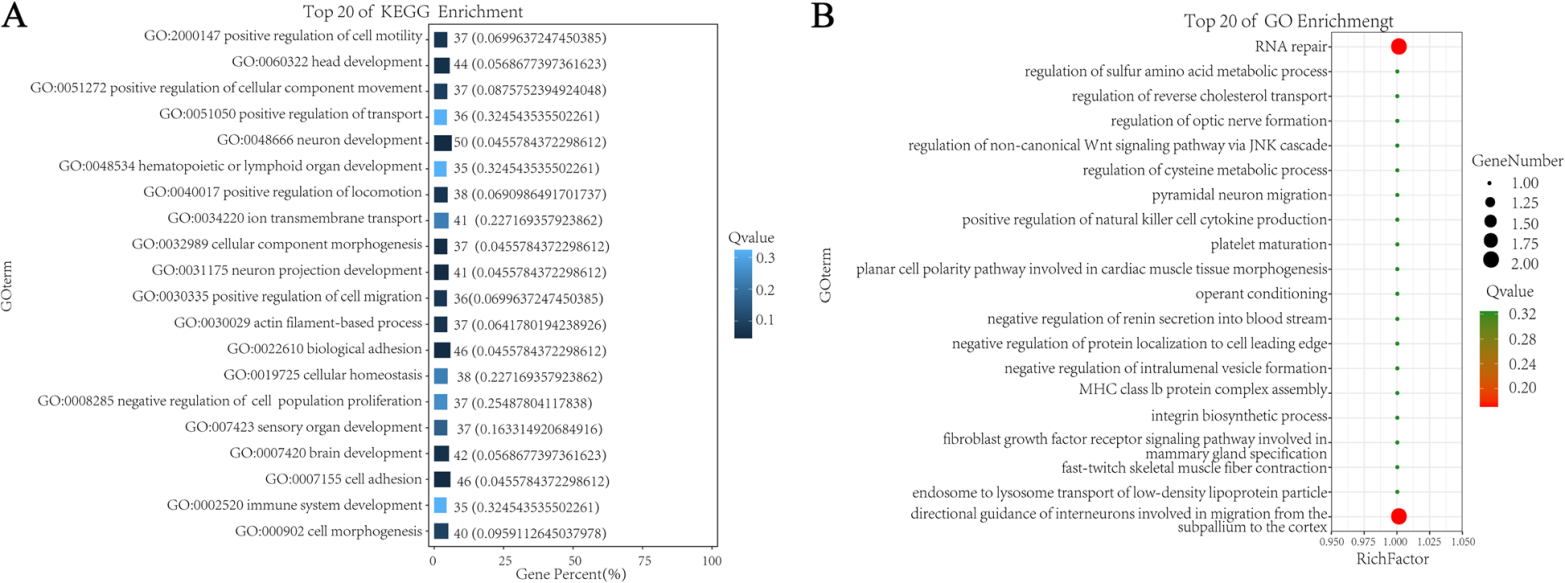
GO enrichment results of biological processes

The GO functional annotation results of candidate genes were shown in Fig. 3. A total of 25 genes were enriched in terms of biological process, and 572 genes were enriched in cellular process. In terms of 8 terms enriched to Cellular Component, organelle was enriched to 341 genes at most. In terms of Molecular Function, a total of 10 terms are enriched, and the maximum number of binging genes is 403. The GO function of candidate genes was significantly enriched and hypergeometric test was used to find out the significantly enriched GO entries. The pathways with the most differentially enriched genes in cell components are multivesicular body, internal vesicle and mitotic spindle assembly checkpoint MAD1-MAD2 complex. The path in which GO term accounts for the largest percentage of all differences is GO:0043005 neuron projection (Fig. 4). The TOP 20 pathways in molecular function have only one gene with a Rich Factor of 1, among which the pathway with GO term accounting for the largest percentage of all differences is GO:0016887ATPase (Fig. 5). The pathways with the most differentially enriched genes in biological processes were RNA repair and subpallium to the cortex and the pathway with GO term accounting for the most percentage of all differences was GO:0048666 neoron development (Fig. 6).

### KEGG enrichment analysis

**Fig. 7:**
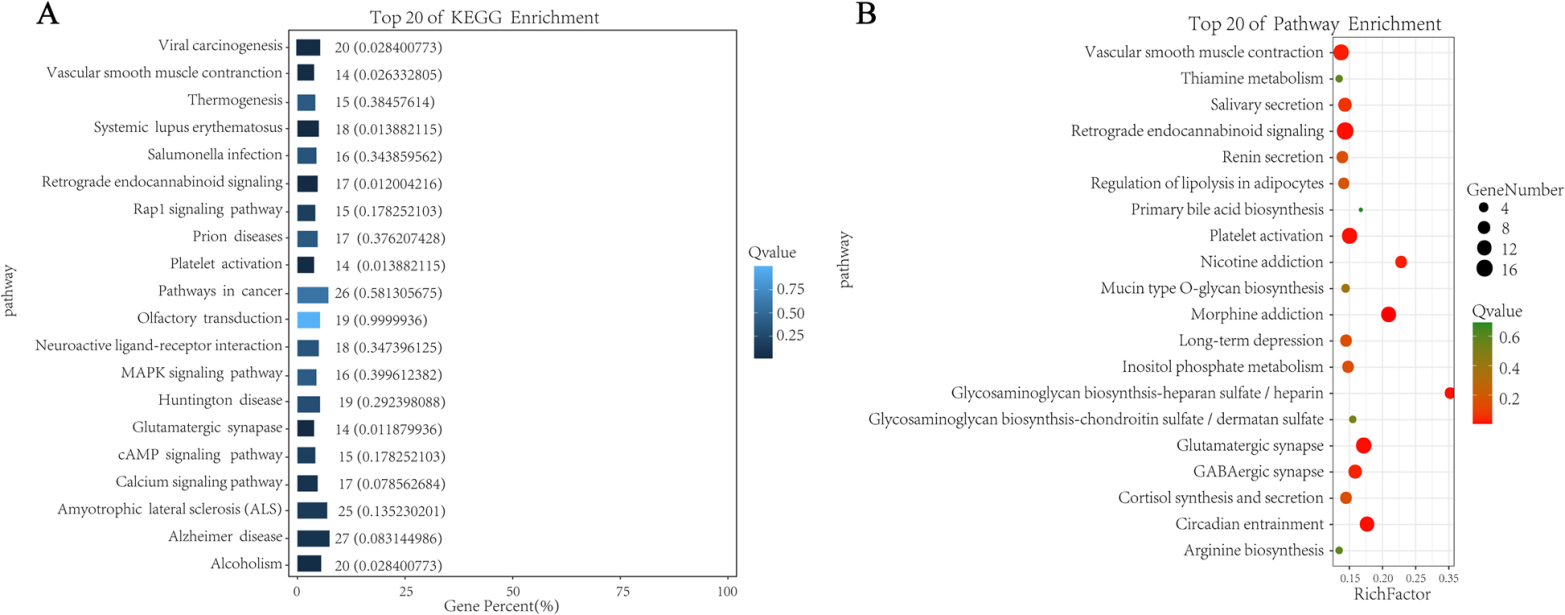
KEGG enrichment analysis

In vivo, different genes coordinate to perform biological functions through the same action pathway. KEGG enrichment analysis is helpful to interpret gene function. Based on the hypergeometric distribution principle, the whole genome genes were taken as background genes, and the candidate genes were analyzed by KO enrichment. As shown in Fig. 7, Among them, Vascular smooth muscle contraction and Retrograde endocannabinoid signaling pathways had the largest number of enriched genes. The Pathways that accounted for the largest percentage of all differences were Alzheimer disease and pathways in cancer.

### Correlation analysis of SNPs in melatonin synthetase and receptor genes

**Fig. 8:**
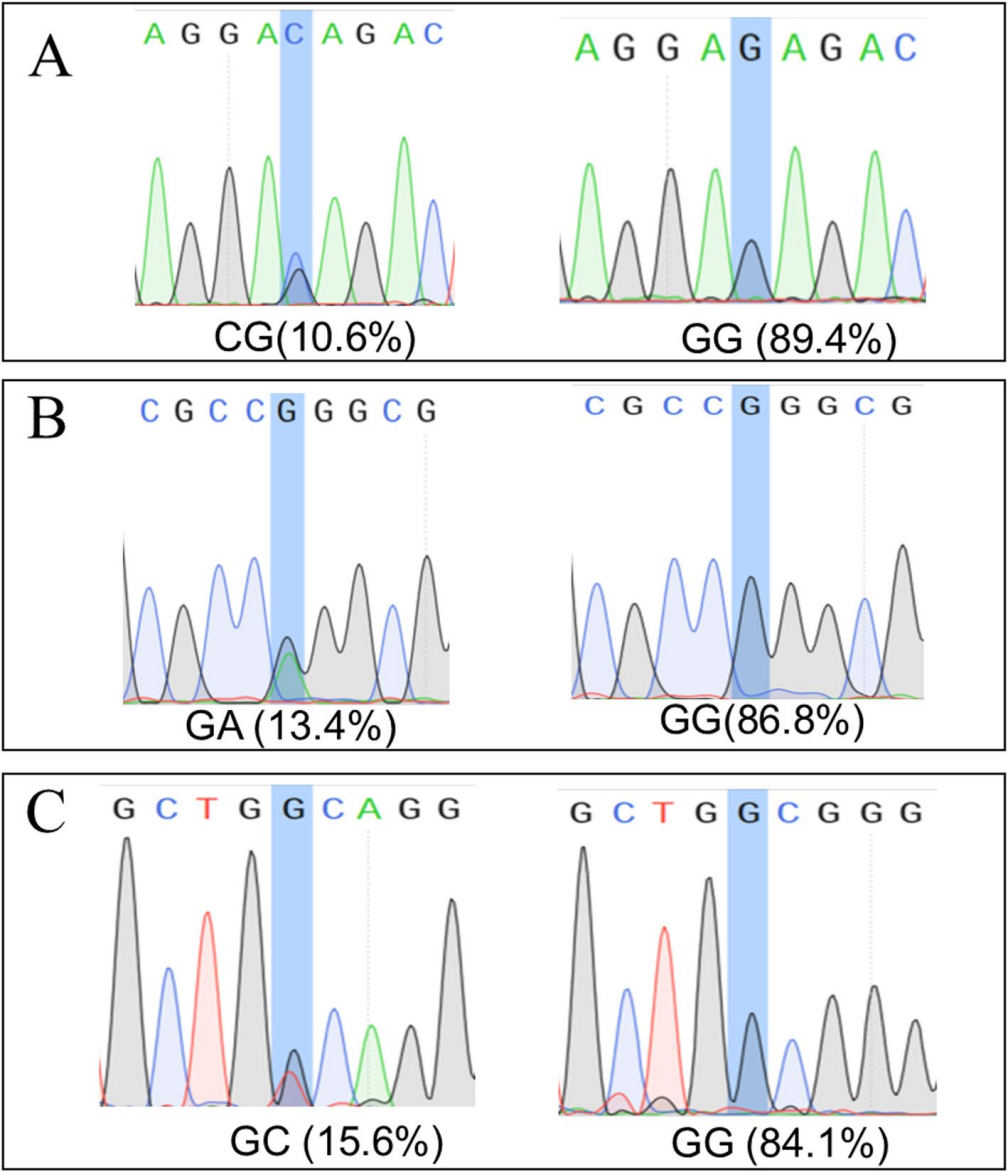
Genotype of significant locus. **A** 7981372 genotype, **B** 7981866 genotype, **C** 17355171 genotype

SNPs of enzyme genes for melatonin synthesis genes of melatonin receptors in high and low melatonin production Hu sheep were counted, among which 14 non-synonymous sites in the exon region had amino acid mutations, as shown in Table 9. R software was used to analyze the correlation between SNP and melatonin concentration in 189 Hu sheep, and it was found that there were three sites with significant correlation with melatonin production (*P* < 0.05) (Table 10), which were ranked 7,981,372, 7,981,866 and 17,355,171, respectively. The levels of melatonin in CG genotype at 7,981,372 site, GA genotype at 7,981,866 site and GG genotype at 17,355,171 site were several folds higher than the average levels in the population (1.64 ng/mL) (Table 11). As shown in Fig. 8, the dominant genotype of SNP 7981372 was GG with a correlation of 89.4% and genotype CG with a correlation of 10.6%. The dominant genotype of SNP 7981866 was GG, and its correlation was 86.8% and 13.4% for GA. The dominant genotype of SNP 17355,171 was GG, and the correlation was 84.1% and 15.6% for GT.

**Table 9:** Summary of non-synonymous mutations

No	Chromosome	Locus	Pre-mutated base	A mutated base	Gene	Amino acid change
P01	NC_040262.1	7,981,745	A	G	AANAT	R/G
P02	NC_040262.1	7,981,372	G	C	AANAT	E/D
P03	NC_040262.1	7,980,810	C	T	AANAT	S/L
P04	NC_040262.1	7,981,866	G	A	AANAT	R/Q
P05	NC_040278.1	604,657	T	G	ASMT	D/A
P06	NC_040272.1	1,684,394	C	T	MTNR1B	R/Q
P07	NC_040272.1	1,684,464	T	C	MTNR1B	I/V
P08	NC_040272.1	1,684,557	T	G	MTNR1B	I/L
P09	NC_040272.1	1,684,269	G	C	MTNR1B	H/D
P10	NC_040277.1	17,354,935	T	C	MTNR1A	I/V
P11	NC_040277.1	17,355,358	C	T	MTNR1A	V/I
P12	NC_040277.1	17,354,943	T	C	MTNR1A	H/R
P13	NC_040277.1	17,377,872	C	G	MTNR1A	A/P
P14	NC_040277.1	17,355,171	G	T	MTNR1A	A/D

**Table 10:** Significance test for mutation sites

AANAT				ASMT	MTNR1B				MTNR1A				
P01	P02	P03	P04	P05	P06	P07	P08	P09	P10	P11	P12	P13	P14
A/G	G/C	C/T	G/A	T/G	C/T	T/C	T/G	G/C	T/C	C/T	T/C	C/G	G/T
0.2074	0.0057	0.0585	0.00084	0.172	0.7849	0.763	0.782	0.3717	0.311	0.0718	0.6461	0.745	0.013

*p* < 0.05 indicates a significant correlation at the 0.05 level and *p* < 0.01 indicates a significant correlation at the 0.01 level

**Table 11:** Melatonin concentrations at significant loci genotypes

Site	P02/7981372		P04/7981866		P14/17355171	
genotype	GG	CG	GG	GA	GG	GT
melatonin	1.43	3.45	1.36	3.52	1.71	1.31

The unit is ng/mL

## Discussion

Due to the nutritional value and the rich taste, the demanding of mutton product has excessed its production globally. To increase mutton production and quality has become an urgent agenda not only for sheep husbandry but also for researchers. It appears that the traditional sheep breeding methods cannot satisfy consumers’ demanding, therefore, the animal breeding techniques have shifted from traditional cross breeding to genome selection which can directly target the traits to be selected [24]. In the current study, we analyzed SNPs related to the production traits of melatonin in Hu sheep. The rationale is that high melatonin content in sheep is often related to high breading ability of this species. It has been reported that melatonin regulates animal reproductive activity and improve oocyte [25] and embryo developmental quality [26]. The transgenic *AANAT* and *ASMT* overexpressed sheep has increased the number of their offspring [27, 28]. Melatonin is secreted mainly by mitochondria, virtually, all cells containing mitochondria have capacity to synthesize melatonin [29, 30]. Melatonin has a wide range of biological functions. It not only regulates animal reproductive activities, but is also a strong antioxidant, which can delay reproductive aging and have anti-tumor activity [31, 32].

Jiang et al. had conducted whole gene sequencing correlation analysis on the body size of Hu sheep and found that 5 SNPs were correlated with body height traits and 4 SNPs were correlated with chest circumference traits [33]. In this study, first, we evaluated the serum melatonin levels in 195 Hu sheep and to identify the distribution of high and low melatonin production individuals, then, the whole genome was sequenced to find the SNPs which are associated with melatonin production traits. Since melatonin is secreted rhythmically, and the levels of melatonin in individuals vary in seasons, light exposure and age [34]. To avoid influences of these factors the environmental information of these sheep exposed have been recorded in detail in advance of a month before the study. It was found that among 195 Hu sheep, 53 individuals (27.2%) were distributed in the high melatonin population with more than 1 ng/mL melatonin and 100 (51.3%) were in low melatonin population with 0–0.5 ng/mL melatonin.

The 7,694,759 SNPs were obtained from 3 individuals with highest and 3 individuals with lowest melatonin productions. SNPs mainly occur in the intronic and splicing regions of the genome. A total of 68,673 SNPs were selected by ED association analysis and 1126 genes were involved in these SNPs. GO and KEGG enrichment analyses showed that the SNPs associated with melatonin traits in Hu sheep at the genome-wide level were enriched into multiple genes in multiple pathways.

*AANAT* and *ASMT* are two key genes in melatonin synthesis and *MTNR1A* and *MTNR1B* are two key genes of melatonin receptors in mediation of melatonin biological functions [2]. Therefore, the analysis was focused on SNPs in the exon region of these four genes and their mutations may lead to amino acid changes and affect melatonin production. Yao et al. have found 3 SNPs of *AANAT* and 4 SNPs of *ASMT* in Holstein dairy cows that can significantly affect the production of melatonin [35]. Here, a total of 14 non-synonymous mutation sites were identified in these four genes. Among them, three sites were found to be significantly correlated with melatonin production in Hu sheep, which were ranked 7,981,372, 7,981,866 and 17,355,171, respectively. The melatonin levels of CG genotype 7,981,372, GA genotype 7,981,866 and GG genotype 17,355,171 were higher (around 1.64 ng/mL) than the average melatonin concentration of (0.5–1.0 ng/mL) in this population. These results indicate that mutations in the three SNPs significantly affect the expression level of melatonin. Melatonin can be considered as an important reproductive regulator. Via hypothalamus and pituitary axis, it can indirectly regulate the seasonal reproductive activities of seasonal breeders [36]. The reproductive system locally produced melatonin including ovary, oocytes, embryo and placenta can directly facilitate the embryo development and increase the numbers of the offspring in many species [37]. The 3 SNPs which occur in the *AANAT*/*ASMT* and *MTNR1A* may improve the enzyme activity to directly enhance melatonin production or promote receptor function which can positively feed-back melatonin production. Since melatonin level in animals is associated with their reproductive efficiency, these three SNPs can serve as the molecular markers to bread Hu sheep with improved reproductive activity.

## Conclusions

In this study, we focused on the of melatonin production ability and its association with SNPs in melatonergic system including its synthesis enzymes and receptors in Hu sheep. By use of the whole-genome sequencing analysis the 3 SNPs located in the *AANAT/ASMT* and *MTNR1A* were identified to directly associated with high melatonin production in the Hu sheep. Considering the positive relationship between melatonin production and reproductive performance in different ruminants, these three SNPs can be selected as the potential molecular markers for breading Hu sheep with the suitable reproductive traits.

### Supplementary Information

**Additional file 1.**

**Additional file 2.**

**Additional file 3.**

**Additional file 4.**

## Data Availability

Whole genome sequencing data of dairy goats have been successfully submitted to the National Center for Biotechnology Information. SRA data: PRJNA942456. KEGG Copyright Permission: 231,015.

## Abbreviations

SNP: Single nucleotide polymorphism
BSA: Bulked segregant analysis
ED: Euclidean Distance
*AANAT*: Aralkylamine N-acetyltransferase
*ASMT*: Acetylserotonin O-Methyltransferase
FSH: Follicle-stimulating hormone
LH: Luteinizing hormone
QTL: Quantitative trait locus
LC–MS/MS: Liquid chromatography tandem mass spectrometry
PE: Paired terminal
PF: Filter Data
fq: FASTQ
