# Supplementary figures and images for: Investigation of SNP markers for the melatonin production trait in the Hu sheep with bulked segregant analysis
Source: BMC Genomics. 2023 Aug 30;24:502. doi: 10.1186/s12864-023-09494-z (PMC10466869; doi:10.1186/s12864-023-09494-z)

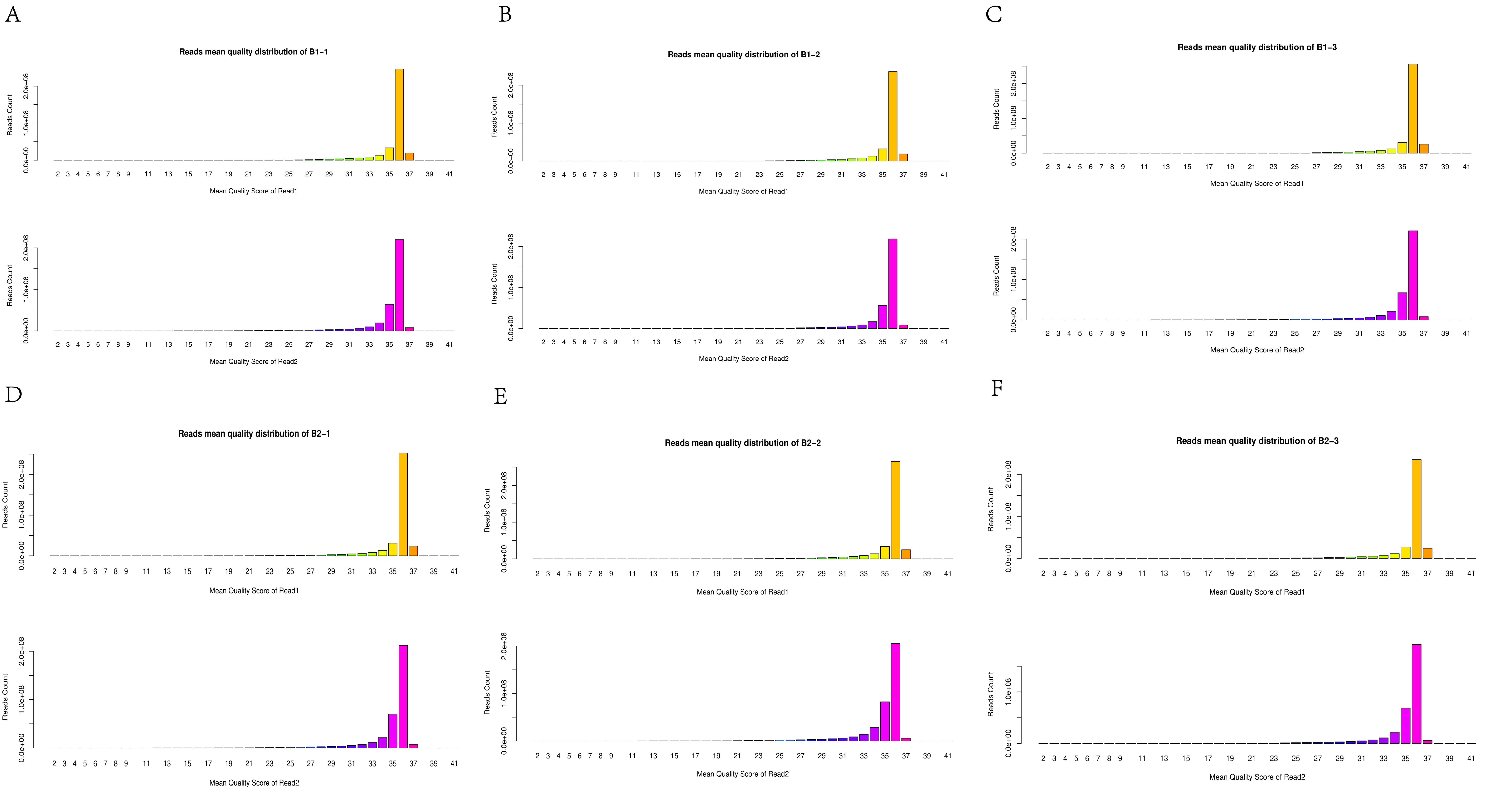

Supplement: Supplementary file 2 — Additional file 2. [file 12864_2023_9494_MOESM2_ESM.tif]

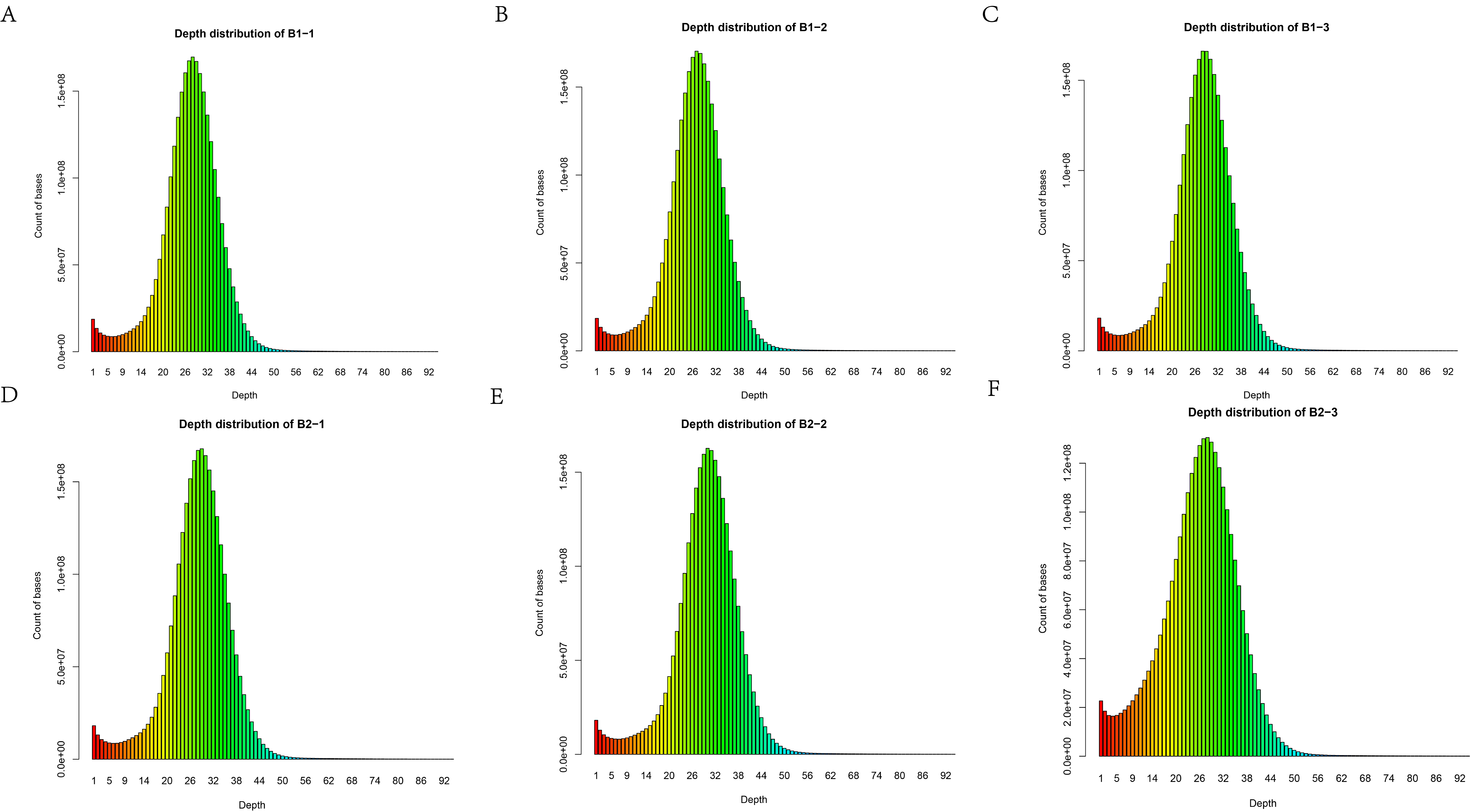

Supplement: Supplementary file 3 — Additional file 3. [file 12864_2023_9494_MOESM3_ESM.tif]

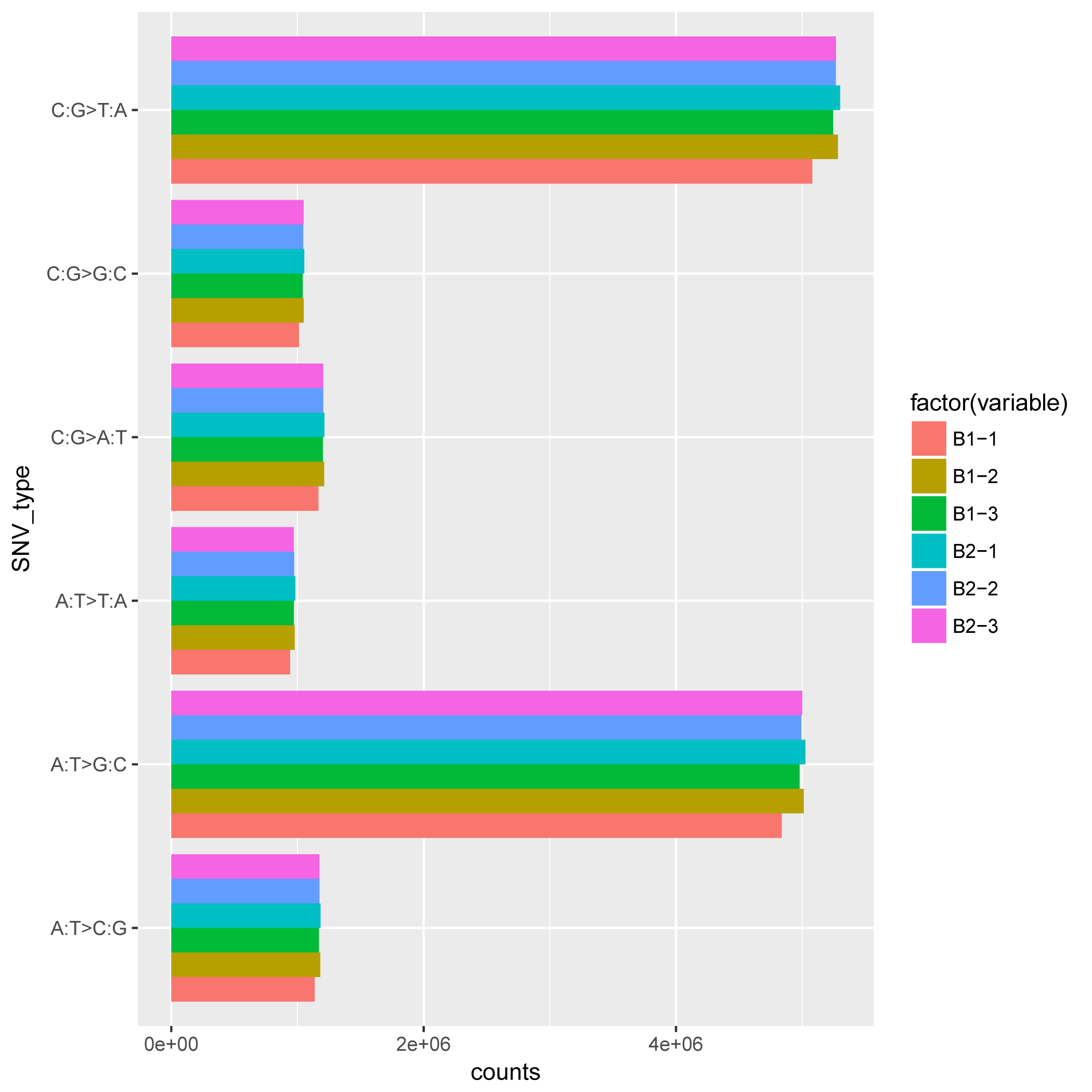

Supplement: Supplementary file 4 — Additional file 4. [file 12864_2023_9494_MOESM4_ESM.tif]
